# Transactions Medical Association of Alabama, April, 1877

**Published:** 1877-10-20

**Authors:** 


Transactions Medical Association of Alabama, April, 1877.—Notice of the above made for our June number, was crowded out and eventualy overlooked. It is a neat and well gotten up volume of 187 pages, containing many valuable and interesting papers. It speaks well for the Profession in Alabama, which indeed, can boast of as large a proportion of talented and progressive men as can be found in any State in the Union.

